# Closure of a giant gastroesophageal mucosal tear after peroral endoscopic myotomy with over-the-scope clip and cyanoacrylate

**DOI:** 10.1055/a-2055-1160

**Published:** 2023-04-11

**Authors:** Oscar Víctor Hernández Mondragón, Luís Fernando García Contreras

**Affiliations:** Division of Endoscopy, Specialties Hospital, National Medical Center Century XXI, Mexico City, Mexico

Insufflation-associated adverse events after peroral endoscopic myotomy (POEM) are frequent although innocuous in most of the patients 1 2 . However, mucosal injuries could lead to perforation, mediastinitis, or bleeding that could be potentially fatal if not treated appropriately. Hemoclips, over-the-scope (OTS) clips 3 , fully covered self-expandable metal stents (FCSEMSs) 4 , and cyanoacrylate 5 have been used as alternative treatments for these cases.

A 63-year-old woman with type II achalasia and an Eckardt score of 10 underwent a POEM procedure without complications. The patient was discharged at 48 hours on a liquid diet. However, at 72 hours she presented to the emergency department with nausea, hematemesis, tachycardia, retrogastric pain, and respiratory distress. An esophagogastroduodenoscopy (EGD) was performed after 3 hours showing a giant 6-cm esophagogastric tear including the distal esophagus, esophagogastric junction (EGJ), and 2 cm of the gastric side ( Fig. 1 ). Mechanical cleaning of clots and debris was performed. A gastrocutaneous-type OTS clip (Ovesco Endoscopy AG, Tübingen, Germany) was placed at the gastric side in retroflexion view and two more in the distal esophagus. Completion of the closure was performed with the injection of 1 cc of undiluted cyanoacrylate (Histoacryl; B-Braun Surgical, Tübingen, Germany) in the distal esophagus in frontal view and 1 cc in retroflexion view at the gastric side ( Fig. 2 ). Dehiscence of the entry site was observed and closure completed with hemostatic clips (Boston Scientific, Marlborough, Massachusetts, USA).

**Fig. 1 a:**
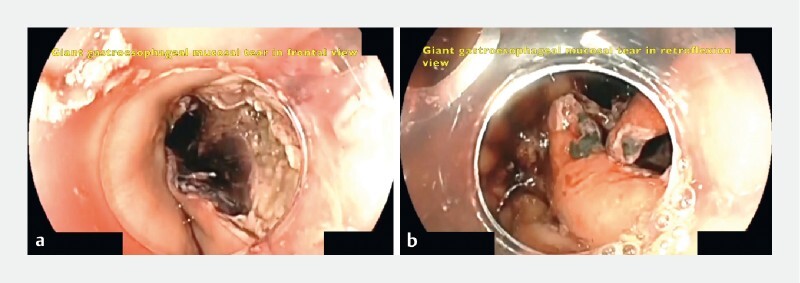
Beginning of esophageal tear at distal esophagus. **b** Distal component including esophagogastric junction and gastric side.

**Fig. 2 a:**
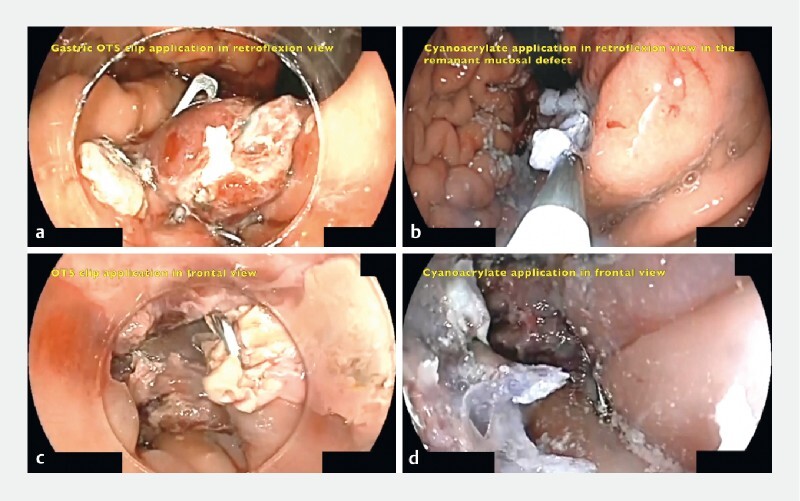
Over-the-scope (OTS) clip placement in retroflexion view. **b** Cyanoacrylate placement at remanent mucosal defect. **c** OTS clip placement in frontal view at distal esophagus. **d** Complementary cyanoacrylate application in distal esophagus mucosal tear.

The patient was admitted to the intensive care unit (ICU) for observation and managed conservatively. Resolution of the systemic inflammatory response syndrome was observed at 48 hours after treatment. A second EGD was performed on the third day showing adequate closure, and a water-soluble contrast esophagram on the fifth day showed adequate passage of contrast without leakage and the OTS clips in place. A liquid diet was started, and the patient discharged on the seventh day. Diet was progressed to a soft and then normal diet in the next 2 weeks.

A follow-up EGD was performed at 8 weeks showing a complete repair of the mucosal tear with the OTS clip in place and clinically an Eckardt score of 0 ( Fig. 3 , Video 1 ).

**Fig. 3 a:**
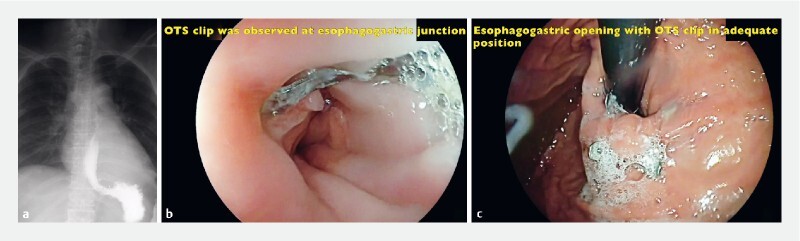
Water-soluble contrast esophagram showing OTS clips in adequate place and no leakage on fifth day after placement. **b, c** OTS clips in place after 8 weeks of closure.

**Video 1** Closure of a giant gastroesophageal mucosal tear after peroral endoscopic myotomy with over-the-scope clip and cyanoacrylate.


Endoscopy_UCTN_Code_CPL_1AM_2AF

## Notes

Endoscopy E-Videos

https://eref.thieme.de/e-videos
*E-Videos* is an online section of the journal *Endoscopy* , reporting on interesting cases and new techniques in gastroenterological endoscopy. All papers include a high-quality video and are published with a Creative Commons CC-BY license. Endoscopy E-Videos qualify for HINARI discounts and waivers and eligibility is automatically checked during the submission process. We grant 100% waivers to articles whose corresponding authors are based in Group A countries and 50% waivers to those who are based in Group B countries as classified by Research4Life (see: https://www.research4life.org/access/eligibility/ ). This section has its own submission website at https://mc.manuscriptcentral.com/e-videos
